# Different Phenotypes in Pseudodominant Inherited Retinal Dystrophies

**DOI:** 10.3389/fcell.2021.625560

**Published:** 2021-02-05

**Authors:** Imen Habibi, Yosra Falfoul, Hoai Viet Tran, Khaled El Matri, Ahmed Chebil, Leila El Matri, Daniel F. Schorderet

**Affiliations:** ^1^IRO-Institute for Research in Ophthalmology, Sion, Switzerland; ^2^Oculogenetic Laboratory LR14SP01, Faculty of Medicine of Tunis, Hedi Rais Institute of Ophthalmology (Department B), Tunis El Manar University, Tunis, Tunisia; ^3^Hôpital Ophtalmique Jules-Gonin, Unité d'oculogénétique, Lausanne, Switzerland; ^4^Faculty of Biology and Medicine, University of Lausanne, Lausanne, Switzerland; ^5^Faculty of Life Sciences, Ecole Polytechnique Fédérale de Lausanne, Lausanne, Switzerland

**Keywords:** retinal dystrophies, whole exome sequencing, pathogenic variants, pseudodominant inheritance, retinitis pigmentosa

## Abstract

Retinal dystrophies (RD) are a group of Mendelian disorders caused by rare genetic variations leading to blindness. A pathogenic variant may manifest in both dominant or recessive mode and clinical and genetic heterogeneity makes it difficult to establish a precise diagnosis. In this study, families with autosomal dominant RD in successive generations were identified, and we aimed to determine the disease's molecular origin in these consanguineous families. Whole exome sequencing was performed in the index patient of each family. The aim was to determine whether these cases truly represented examples of dominantly inherited RD, or whether another mode of inheritance might be applicable. Six potentially pathogenic variants in four genes were identified in four families. In index patient with enhanced S-cone syndrome in F1, we identified a new digenetic combination: a heterozygous variant p.[G51A];[=] in *RHO* and a homozygous pathogenic variant p.[R311Q];[R311Q] in *NR2E3*. Helicoid subretinal fibrosis associated with recessive *NR2E3* variant p.[R311Q];[R311Q] was identified in F2. A new frameshift variant c.[105delG];[105delG] in *RDH12* was found in F3 with cone-rod dystrophy. In F4, the compound heterozygous variants p.[R964^*^];[W758^*^] were observed in *IMPG2* with a retinitis pigmentosa (RP) phenotype. We showed that both affected parents and the offspring, were homozygous for the same variants in all four families. Our results provide evidence that in consanguineous families, autosomal recessive can be transmitted as pseudodominant inheritance in RD patients, and further extend our knowledge of pathogenic variants in RD genes.

## Introduction

Retinal dystrophies (RD), a group of heterogeneous hereditary diseases, are caused by perturbed photoreceptor function. It presents significant genetic heterogeneity and contribute notably to the etiology of blindness around the world (den Hollander et al., 2010). To date, more than 300 disease associated genes have been implicated in the pathogenesis of the disease, most of which are involved in the development and normal function of photoreceptor and cells from the retinal pigment epithelium (RPE) (den Hollander et al., 2008, 2010). Clinical symptoms vary widely among different RD subtypes and disease genes (Ellingford et al., 2016). RD are generally classified based on the type of photoreceptor cells affected, i.e., rods or cones, and thus on the location, macula or peripheral retina (Nash et al., 2015).

The described modes of inheritance in RD are recessive (ar) or dominant (ad) and autosomal or X-linked. A few rare cases of RD exist, in which mitochondrial mutations, digenic inheritance, and pseudodominant transmission have been documented (Lewis et al., 1999; Liu et al., 2019).

In this study, we used whole exome sequencing (WES) and Sanger sequencing to investigate four consanguineous families with several members affected by RD, inherited in an apparent ad pattern. We aimed to identify potential gene variants underlining these cases and described the genotypic and phenotypic findings in these complicated RD pedigrees. Distinct inheritance patterns and disease-causing variants were identified in all families.

## Materials and Methods

### Ethical Compliance

This study was approved by the Local Ethics Committee of the Hedi Rais Institute in Tunisia. Informed consent was obtained from all participants in the study. Analyses were done in accordance with local guidelines.

### Clinical Investigations

Our study contains four Tunisian families with non-syndromic RD (Table 1). All patients underwent detailed clinical examinations and their family history was collected. A comprehensive ophthalmological examination was performed at the Department B of Hedi Rais Institute of Ophthalmology, Tunis (Tunisia), including best-corrected visual acuity (BCVA), slit lamp, dilated fundus examination, and full-field electroretinography according to the International Society for Clinical Electrophysiology of Vision (ISCEV) standards (Métrovision, France), swept source optical coherence tomography (SS-OCT, Topcon, Swept Source DRI-OCT Triton, Japan), and fundus autofluorescence (FAF) imaging (Heidelberg, HRA 2 Spectralis, Germany).

**Table 1:** Clinical data of four families with gene-associated RD.

**Family**	**Patient**	**Gender**	**Age [years]**	**Age of onset**	**Visual acuity OD/OS/OU**	**Ophthalmoscopy**	**Optical coherence tomography**	**Full-field ERG (OU)**	**Diagnosis**
F1	II.2	F	19	6	1/20 OU	Mid-peripheral nummular pigment clumping and atrophy	Diffuse macular cystoids edema	Similar waveforms under photopic and scotopic conditions	Enhanced S-cone SD
	I.1	F	68	Infancy	LP LP	Diffuse retinal atrophy	Macular atrophy	Extinct response	Enhanced S-cone SD
F2	II.1	M	13	Infancy	3/10 OU	Circumferential fibrosis on the PP	Macular cysts	Similar responses under scotopic and photopic stimulations	Enhanced S-cone SD
	I.1	M	51	10	1/10 OU	Circumferential fibrosis on the PP	Macular cystoid edema	-	Enhanced S-cone SD
F3	II.1	F	13	Under 5	1/10 OU	Macular atrophy, peripheral spicule déposits	-	-	Cone-rod dystrophy
	I.2	F	37	Infancy	LP	Diffuse macular and peripheral deposits	-	-	Cone-rod dystrophy
					LP				
F4	II.1	F	37	Second decade	5/10 OU	Preserved PP, peripheral spicule deposits	Normal	Reduced rod responses	RP
								Preserved cone response	
	II.2	F	32		7/10 OD		Normal	-	RP
					6/10 OS				
	II.3	F	28		6/10 OU		Normal	-	RP
	II.4	F	25		9/10 OU		Normal	-	RP
	I.2	F	63		1/10	Macular atrophy	Macular atrophy	-	Advanced RP
						Spicule deposits			

*OD, right eye; OS, left eye; OU, both eyes; RP, retinitis pigmentosa; F, female; M, male; PP, posterior pole*.

### Genetic Analysis

Peripheral blood samples were obtained from 4 index patients and 13-related individuals, including parents and affected siblings. DNA was extracted from leukocytes according to the salting-out method (Miller et al., 1988).

We sequenced the exome in the index patient, then variants were confirmed by Sanger sequencing and segregation was established in all families.

#### Whole Exome Sequencing (WES)

Next-generation sequencing was done at Sophia Genetics (SOPHiA GENETICS SA, Saint-Sulpice, Switzerland) using the clinical exome solution v.2 (CES2). The complete list of genes analyzed in CES2 can be obtained at www.sophiagenetics.com. ~98.95% of target regions were covered at least 25×.

#### Variant Assessment

All variants were first filtered against several public databases for the minor allele frequency (MAF) of <1%. dbSNP database served as a reference to exclude any known frequent variants occurring in coding (missense) and regulatory regions including splice site and promoter regions. Table 2 lists the criteria to classify the variants described here.

**Table 2:** Classification of the identified variants.

**Variant ID**	**Evidence of pathogenicity**	**Class**
*RHO*: p.G51AS	BS1, BS2, BP2	Likely benign
*NR2E3*: p.R311Q	PS3, PM1, PP5, BS1	Likely pathogenic
*RDH12*:c.105delG	PVS1, PM1, PM2, PM3, PP1	Pathogenic
*IMPG2*:p.R964^*^	PVS1, PM2, PP3, PP5	Likely pathogenic
*IMPG2*:p.W758^*^	PVS1, PM2, PP3, PP5	Likely pathogenic

A variant was considered novel if it has not been described in the medical literature or was not present in the Human Mutation Database (www.hgmd.cf.ac.uk/ac). The variant frequency in the control population was evaluated using gnomAD (http://gnomad.broadinstitute.org/).

#### Sanger Sequencing

All variants were confirmed by Sanger sequencing. PCR reactions and amplification conditions were performed using the following primers in Table 3.

**Table 3:** Primers.

**Primers**	**Forward**	**Reverse**
*RHO* exon 1	TCA TCC AGC TGG AGC CCT GAG T	AAC ATT GAC AGG ACA GGA GAA GG
*NR2E3* exon 6	TCT GAG CCT CTG GCT GAT GTC A	AGA AGG GAG TCC AGC CTC AC
*IMPG2* exon13	TGC CCA TCT TCG CAG ATA CT	TCC AAA CTC TCT CTG ATT CTG G
*IMPG2* exon14	GGA AAA GTG AGG CAG GGT CT	TGG GTA GAG AAA GGA ATG GAG G
*RDH12* exon4	CTT AGT GTG AGC TCG TGA AGG A	TTG GAC TTG AAT CCC AGG TT

## Results

### Clinical Diagnosis

#### Family 1

**Figure 1:**
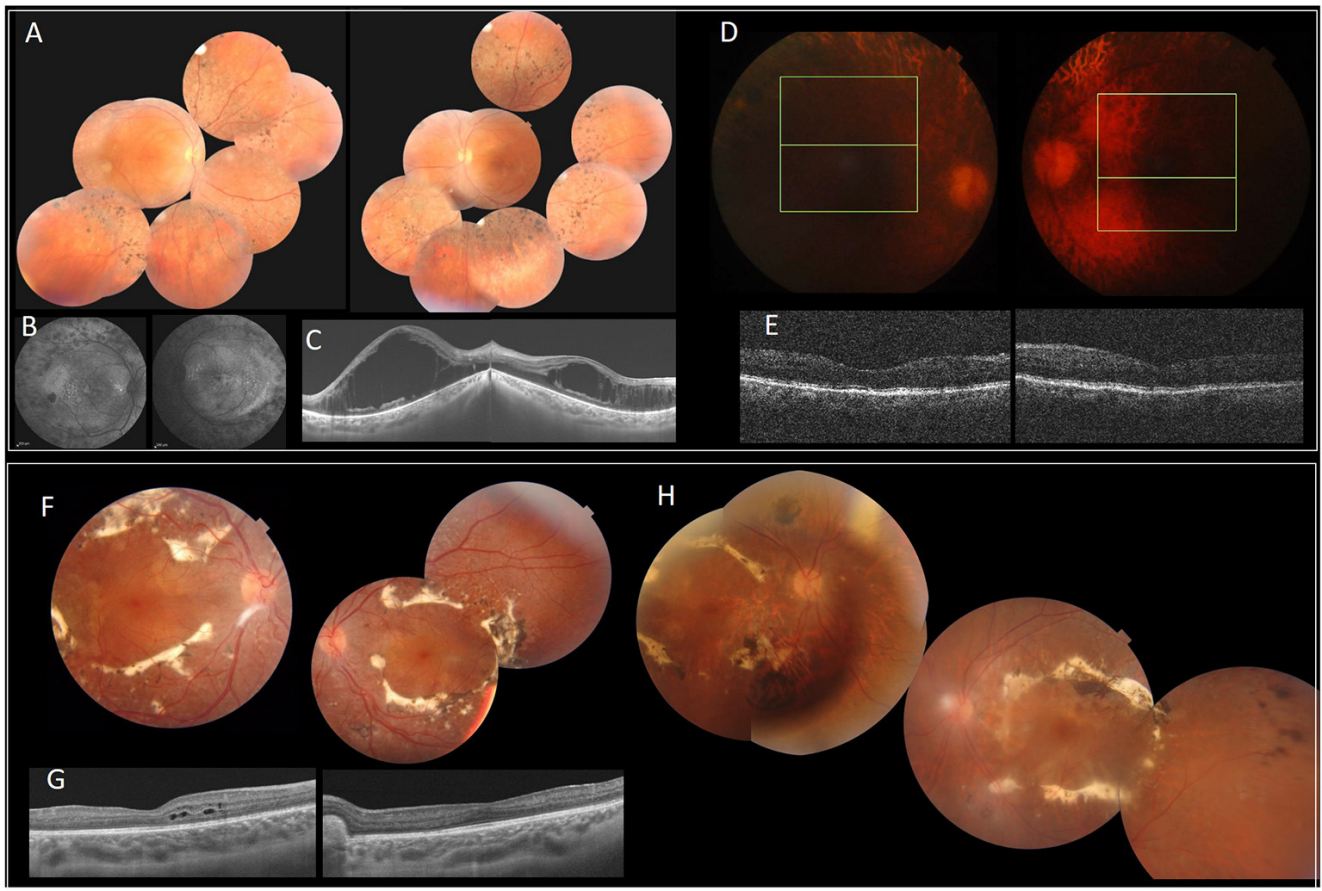
Clinical and imaging features of patients with *NR2E3* mutations. F1 **(A–E)**. **(A)** FP of the index patient with peripheral nummular spicule deposits and atrophy. **(B)** FAF with peripheral hypo-autofluorescence spots and heterogeneous autofluorescence of the posterior pole. **(C)** SS-OCT. Bilateral cystoid macular edema. **(D,E)** Diffuse retinal atrophy. F2 **(F–H)**. **(F,H)** FP of the index patient (II.1) **(F)** and his father (I.1) **(H)** with circumferential fibrosis and nummular pigment clumping and peripheral spicule deposits. **(G)** SS-OCT. intraretinal cysts (II.1). FP, Fundus photography.

**Figure 2:**
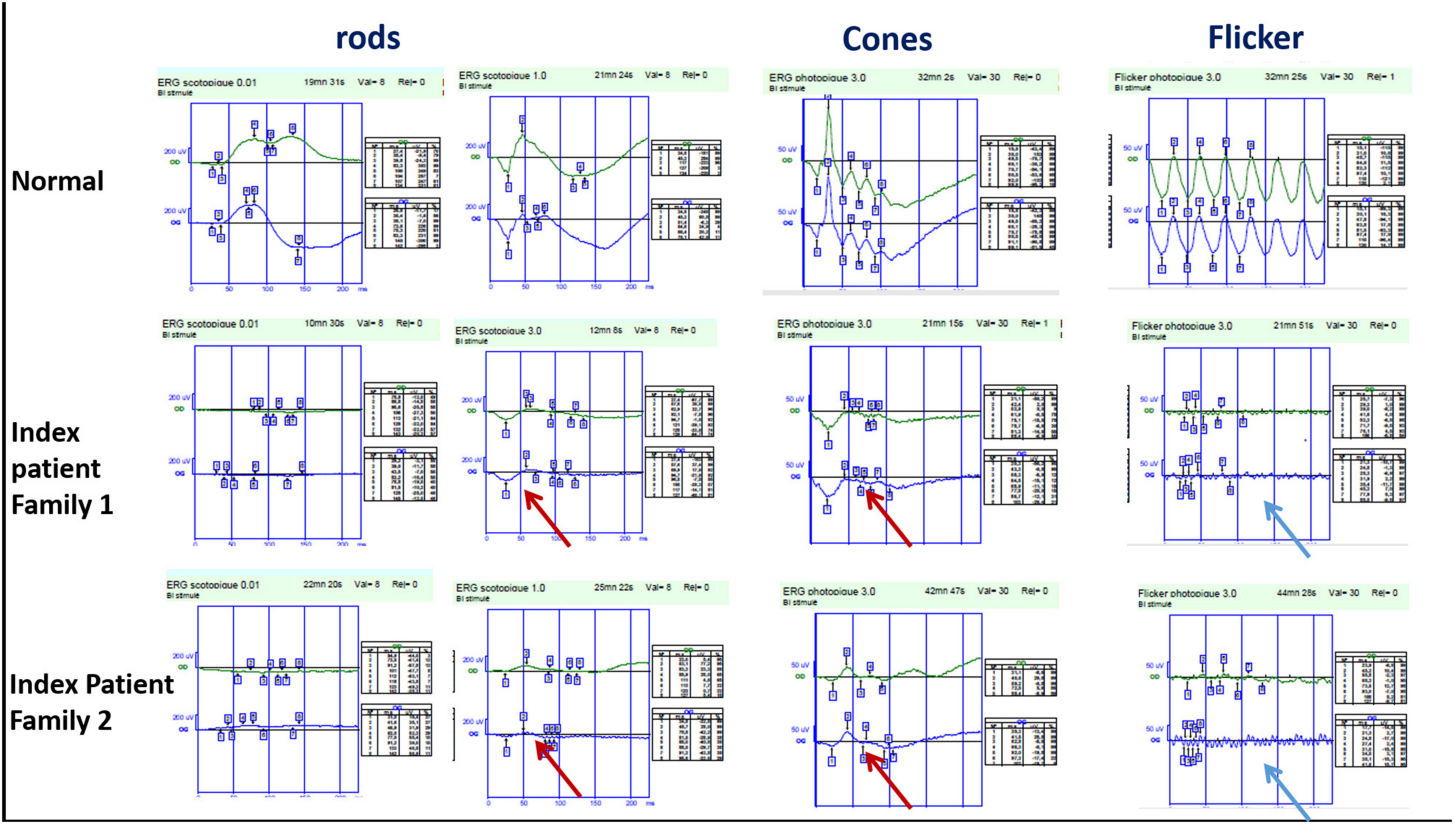
Full-field ERG findings in enhanced-S cone syndrome patients from families 1 and 2 showing similar waveforms in both scotopic and photopic conditions (red arrow) with markedly reduced flicker (blue arrow).

The index patient is a 19-year-old girl from consanguineous marriage with the diagnosis of enhanced S-cone syndrome. She had nyctalopia since the age of 6 years along with visual impairment. Her BCVA was limited to 1/20 in both eyes (OU). On fundus examination, she had mid-peripheral nummular pigment clumping, atrophy and cystoid macular edema better visualized on SS-OCT. FAF showed hyper FAF dots in the posterior pole (Figures 1A–C). In full-field ERG, reduced cone and rod responses with similar waveforms under photopic and scotopic conditions were detected (Figure 2). Affected mother had severe visual impairment with cataract and diffuse retinal atrophy (Figures 1D,E).

#### Family 2

The index patient was a 13-year-old boy from consanguineous marriage presenting with retinal dystrophy. His BCVA was limited to 3/10 OU. On fundus examination, he had circumferential fibrosis, nummular pigment clumping on the posterior pole and peripheral spicule deposits with preserved macular region (Figures 1F,G). In full-field ERG, there were similar cone and rod responses under photopic and scotopic conditions with markedly reduced flicker, which allowed us to retain the diagnosis of enhanced S-cone syndrome (Figure 2). Affected father had similar retinal phenotype with bilateral cataract (Figure 1H).

#### Family 3

**Figure 3:**
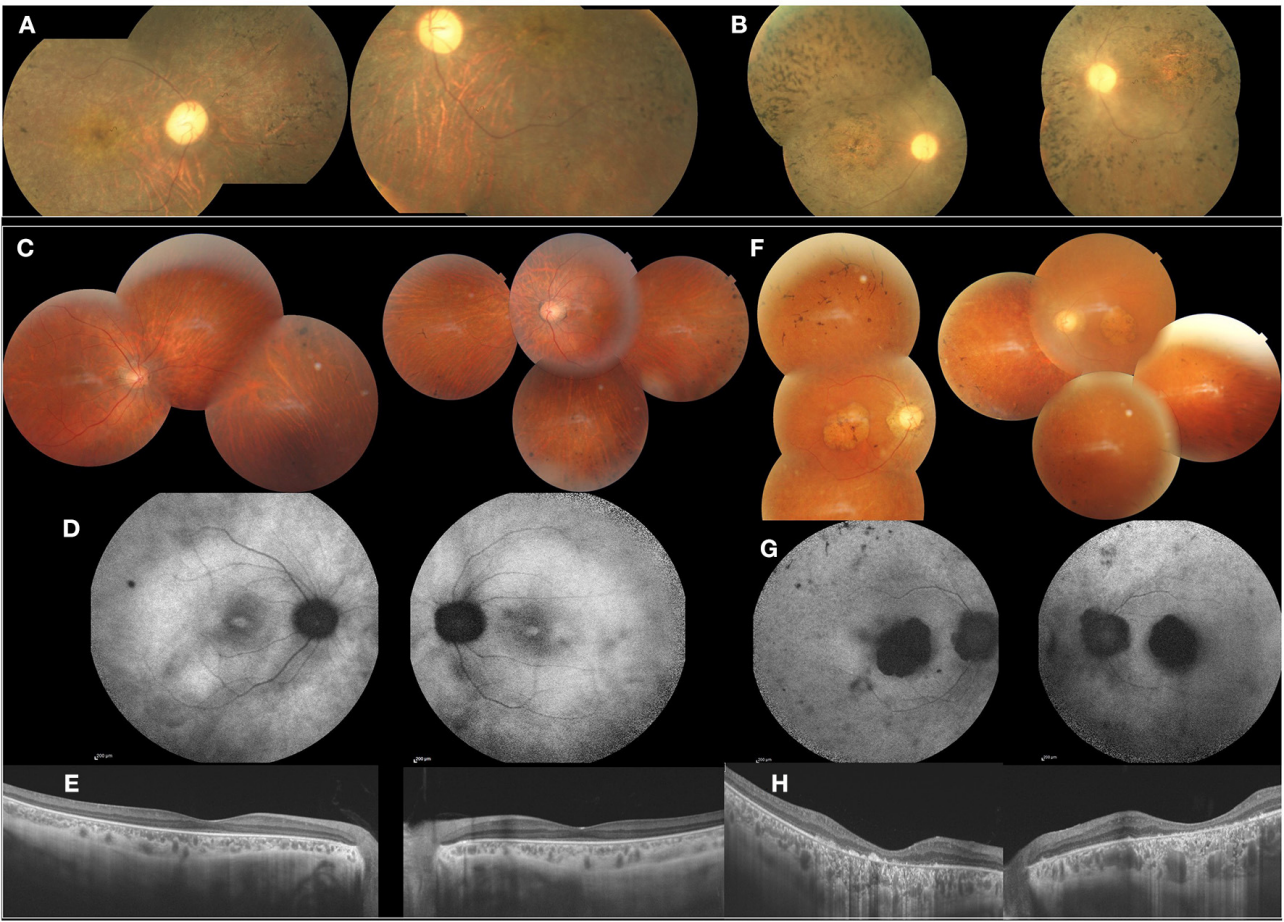
Clinical and imaging features of affected patients from F3 and F4, F3 **(A,B)**. FP of the index patient (II.1) and her mother (I.2) showed cone-rod dystrophy with macular atrophy and spicule deposits in the mid peripheral retina. F4 **(C–H)**. **(C)** FP of the index patient (II.2) with peripheral spicule migrations and preserved posterior pole. **(D)** Foveolar hyperautofluorescence, peripheral hypo-autofluorescence spots. **(E)** SS-OCT. Preserved retinal macular layers. **(F)** FP of the mother (I.2) showing macular atrophy and peripheral spicule deposits. **(G)** FAF with lack of autofluorescence in the macula. **(H)** SS-OCT. Severe and diffuse macular atrophy.

The index patient is a 13-year-old girl with impaired visual acuity (BCVA: 1/10 OU) and nyctalopia since her first decade of life. She had cone-rod dystrophy with macular atrophy and spicule deposits in the mid peripheral retina (Figure 3A). In full-field ERG, there were reduced cone and rod responses. Her mother had similar phenotype with BCVA limited to light perception and diffuse spicule deposits in fundus examination (Figure 3B).

#### Family 4

The index patient is a 38-year-old male diagnosed with familial retinitis pigmentosa (RP). BCVA was 5/10 s. On fundus examination, there were peripheral spicule migrations and preserved posterior pole (Figures 3C–E). The same phenotype was present in three-affected sisters. The mother, had BCVA limited to 1/20 OU with macular atrophy and peripheral spicule deposits (Figures 3F–H).

### Molecular Diagnosis

#### Identified Pathogenic (P) and Likely Pathogenic (LP) Variants

**Figure 4:**
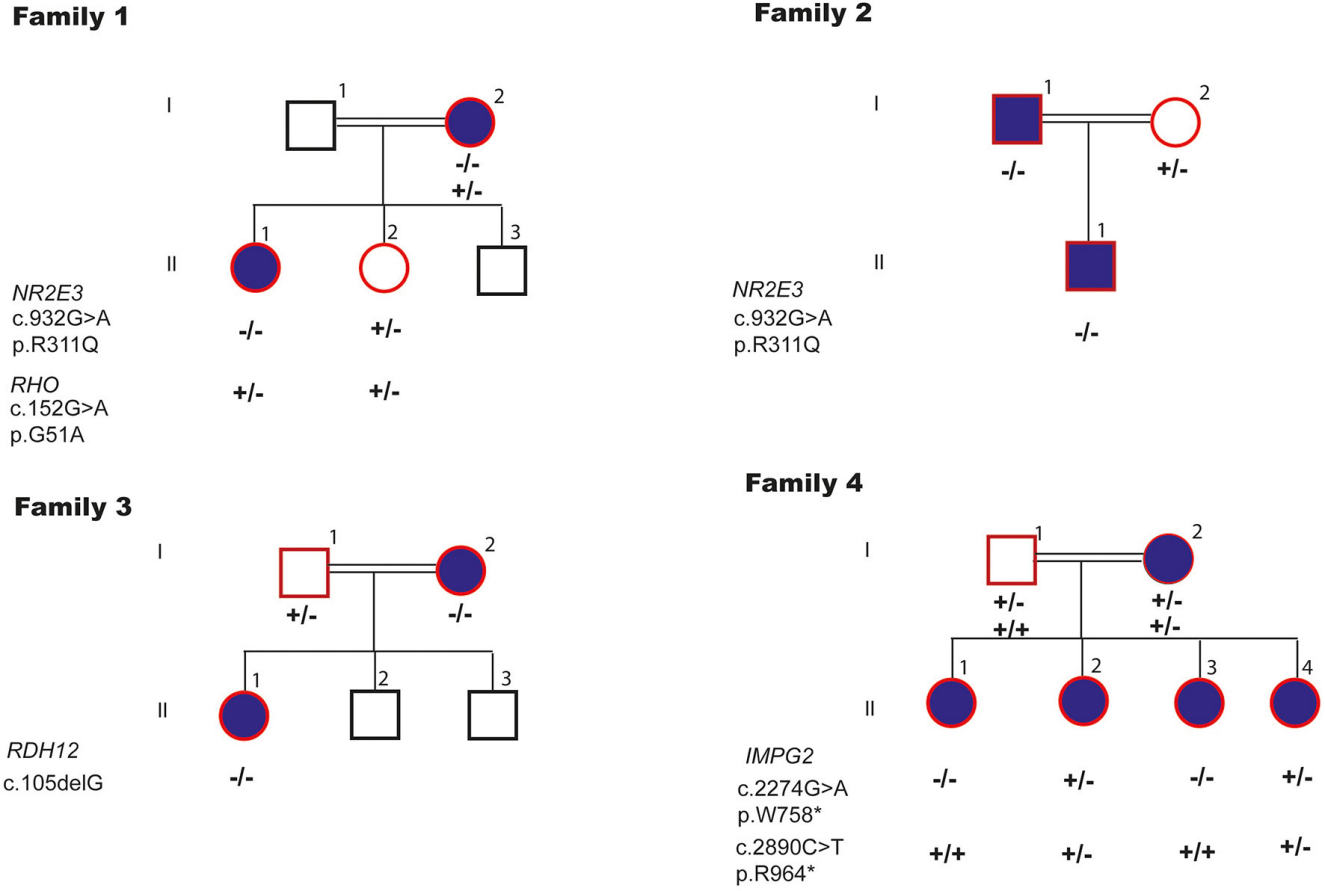
Segregation of the pathogenic variants in four genes identified in four families.

Six potentially pathogenic variants in four genes were identified in four families (Figure 4).

In the index patient with enhanced S-cone syndrome in F1, we identified, a new digenetic combination: a heterozygous likely benign variant p.[G51A];[=] in *RHO* and homozygous LP variant p.[R311Q];[R311Q] in *NR2E3*. Helicoid subretinal fibrosis associated with a recessive *NR2E3* variant p.[R311Q];[R311Q] was identified in F2. New frameshift P variant c.[105delG];[105delG] in *RDH12* was found in the index patient of F3 with cone-rod dystrophy (CRD). Compound heterozygous LP variants p.[R964^*^];[W758^*^] in *IMPG2* were observed in the index patient with RP in F4. We have shown that both, the affected parents and the offspring, are homozygous for the same variant in the four families. These variants are classified as pathogenic according to the American College of Medical Genetics (ACMG) guidelines.

## Discussion

In this report, molecular testing revealed the coexistence of pathogenic variants affecting distinct RD causing genes in four RD families. Among the five pathogenic variants identified in these families, one was novel (*RDH12* c.105delG, p.Q36Sfs^*^6) and four were recurrent (*RHO* p.G51A, *NR2E3* p.R311Q and *IMPG2* p.W758^*^, p.R964^*^) (Macke et al., 1993; Haider et al., 2000; Bandah-Rozenfeld et al., 2010; Patel et al., 2016).

Two families, F1 and F2, carried the most frequent variant p.R311Q in *NR2E3*. Gerber et al. have previously identified this in patients who may have Enhanced S-cone syndrome (ESCS) (Gerber et al., 2000). Later, Haider et al. reported the same p.R311Q variant with 44.8% frequency in patients with ESCS (Haider et al., 2000) and it was also reported in a patient with Goldmann-Favre syndrome (GFS) (Bernal et al., 2008). The particularity in F1 is the presence of a second heterozygous variant p.G51A in *RHO*. Two diseases are associated with *RHO* variants, RP and congenital stationary night blindness (CSNB). However, in F1 the index patient presented with ESCS, which is more related to *NR2E3*. Interesting, and although *RHO*: p.G51A has been involved in retinitis pigmentosa (Macke et al., 1993), GnomAD mentions a frequency of 0.1% in the general population and ClinVar classifies this variant as benign/likely benign. Family F1 would favor the latter classification.

New pathogenic homozygous variant c.[105delG];[105delG], p.[Q36Sfs^*^6;[Q36Sfs^*^6] in *RDH12* was detected in F3. Variants in *RDH12* have been previously linked to Leber congenital amaurosis (LCA) and ad-RP. Retinal pathologies resulting from variants in *RDH12* gene can be inherited in either ad or ar mode (Kumaran et al., 2017; Sarkar et al., 2020). Ar biallelic variants in *RDH12* were first identified in three consanguineous Austrian families with severe retinal dystrophy (Janecke et al., 2004).

F4 harbored compound heterozygous variants p.[W758^*^];[R964^*^] in *IMPG2*. This is the first time the association of these two variants is described. Biallelic variants in *IMPG2* have been shown to underlie recessive childhood-onset rod-cone dystrophy with early macular involvement in several families (Khan and Al Teneiji, 2019). At the heterozygous state, *IMPG2* variants have been associated with dominant vitelliform macular dystrophy (Brandl et al., 2017). In F4, the affected mother carried compound likely pathogenic variants, the unaffected father carried a p.W758^*^ heterozygous variant and the affected children a combination of these variants. Pseudodominant inheritance occurs when an individual with a known recessive disorder has a clinically unaffected partner, but then unexpectedly gives birth to children who are affected with the same recessive disorder as the affected parent (Thompson and Thompson, 1986). Although all the parents in the described families were first cousins, each disease followed a dominant pattern of inheritance, with one affected parent and one or several affected children. Molecular analyses allowed us to correctly classify the mode of inheritance as pseudo-dominant, a mode usually associated with a high mutant frequency, like in hemochromatosis.

## Data Availability Statement

The datasets presented in this study can be found in online repositories. The names of the repository/repositories and accession number(s) can be found in the article/supplementary material.

## Ethics Statement

The studies involving human participants were reviewed and approved by Local Ethics Committee of the Hedi Rais Institute in Tunisia. Written informed consent to participate in this study was provided by the participants' legal guardian/next of kin. Written informed consent was obtained from the individual(s), and minor(s)' legal guardian/next of kin, for the publication of any potentially identifiable images or data included in this article.

## Author Contributions

DS and LE designed the research protocol. IH and DS performed the analyses. YF, HT, KE, AC, and LE performed the clinical evaluation. IH, YF, LE, and DS wrote the manuscript. All authors reviewed the manuscript.

## Conflict of Interest

The authors declare that the research was conducted in the absence of any commercial or financial relationships that could be construed as a potential conflict of interest.
